# VibroTouch: Active Tactile Sensor for Contact Detection and Force Sensing via Vibrations

**DOI:** 10.3390/s22176456

**Published:** 2022-08-27

**Authors:** Danissa Sandykbayeva, Zhanat Kappassov, Bakhtiyar Orazbayev

**Affiliations:** 1 Robotics Department, Nazarbayev University, Nur-Sultan 010000, Kazakhstan; 2 Physics Department, Nazarbayev University, Nur-Sultan 010000, Kazakhstan

**Keywords:** active vibration sensing, tactile sensing, contact detection

## Abstract

Accurate and fast contact detection between a robot manipulator and objects is crucial for safe robot–object and human–robot interactions. Traditional collision detection techniques relied on force–torque sensors and Columb friction cone estimation. However, the strain gauges used in the conventional force sensors require low-noise and high-precision electronics to deliver the signal to the final user. The Signal-to-Noise Ratio (SNR) in these devices is still an issue in light contact detection. On the other hand, the Eccentric Rotating Mass (ERM) motors are very sensitive to subtle touch as their vibrating resonant state loses immediately. The vibration, in this case, plays a core role in triggering the tactile event. This project’s primary goal is to use generated and received vibrations to establish the scope of object properties that can be obtained through low-frequency generation on one end and Fourier analysis of the accelerometer data on the other end. The main idea behind the system is the phenomenon of change in vibration propagation patterns depending on the grip properties. Moreover, the project’s original aim is to gather enough information on vibration feedback on objects of various properties and compare them. These data sets are further analyzed in terms of frequency and applied grip force correlations in order to prepare the ground for pattern extraction and recognition based on the physical properties of an object.

## 1. Introduction

Vibrations are undoubtedly vital to us. Without them, for instance, we could not detect events needed to manipulate an object or even walk. Therefore, vibration sensing has attracted the attention of scientists since the era of Leonardo da Vinci, who pioneered the investigation of friction and slippage [1]. Frictional resistance between object and hand lets us grasp an object when we apply enough force at the contact points [2]. This force prevents the body from slipping and, therefore, from falling. Even though we may grasp objects—when volumetric boundaries of a body fit into the hand—with form closure [3], true dexterity appears when we grab and manipulate objects with force closure [2]. We finely control both the fingertip motions [4] and the forces at the points of contact with a grasped object to prevent its slippage [5]. Thanks to the sense of touch in our fingers that can detect friction-induced vibrations [6,7,8], these forces can increase almost immediately if the object starts to slip. Indeed, a person’s visual acuity cannot affect the ability to manipulate. On the other hand, the absence of touch sense—through anesthetizing the fingertips as shown in the experiments on lightning-up matches [9]—dramatically impairs object manipulation capability.

Humans can effortlessly manipulate objects and tools by applying precisely controlled forces. In fact, robot hands are rather crude in terms of manipulation skills compared with humans [10]. The effectiveness of the mechanisms for object manipulation was evaluated in the First Amazon Picking Challenge [11] and the DARPA challenge, which was highlighted in [12]. The human hand can detect contact with an object and prevent the grasped object from falling—thanks to its advanced tactile sensing—by detecting and rapidly correcting applied forces. This is hard to replicate in mechatronic systems, such as robot hands or prostheses, where sensorimotor skills for contact detection and slippage control is more limited compared with real hands [13].

With the aim to close the gap in sensorimotor skills in robots, slip detection systems based on vibrations have been developed by researchers for both prehensile (e.g., grasping an object for a pick-and-place task) and non-prehensile (e.g., pushing an object or typing a keyboard) manipulation. In both cases, tactile signals have been used for detection of the current manipulation phase (**contact/no contact**, slippage, sliding, etc.) [14]. Contact detection was also used for object exploration, recognition, and material classification as surveyed in [15,16,17,18]. Depending on the transduction type of the tactile sensor—it can be dynamic or static according to the time response and may represent an array of data, vector or scalar— a stable grasp can be assessed from contact forces [19], contact pressure profile [20,21], and friction-induced vibrations [22,23].

Traditionally, tactile sensing approaches relied on collision detection techniques, where industrial force–torque sensors are the golden standard [15]. Unfortunately, the microelectromechanical systems (MEMS) used in these conventional force sensors require low-noise and high-precision electronics. Advances in force gauges allowed the miniaturization of these force sensors and led to better sensing performance. Nevertheless, the Signal-to-Noise Ratio (SNR) is still a burden towards light contact detection [24].

In force sensor-based approaches, most approaches are seeking for the stability of a grasp rather than contact detection. The stability is evaluated by the ratio of normal-to-tangential reaction forces and the static coefficient of friction. Slippage avoidance is ensured by maintaining an object within the Coulomb friction cone. The tangential force can be obtained by force–torque sensors [2]. The analogous principle is applied for preventing rotational slippage by the estimation of rotational friction, which is more complex to model than the linear one [25]. In [26], the rotational slippage was leveraged to manipulate a cylinder so that it undergoes a desired motion due to gravity. Other approaches can rely on dynamic friction models that allow the prediction of a slip. For example, using a force–torque sensor installed on a robot hand, [19] estimated the coefficients of the dynamic LuGre friction model of contact with an unknown object through two exploratory motions. The breakaway friction ratio was then computed to predict slippage.

One of the first approaches within this group is described in [27]. The authors detected the loosing of a contact due to slippage by calculating changes in tactile pattern represented by a matrix in which the increase, decrease, and absence of any change in force correspond to values 1, −1, and 0, respectively. Slippage is derived by summing and subtracting the neighbor elements in a 4×4 tactile sensing array attached onto a prosthetic hand. New generations of these sensors have better spatial resolution and more numbers of tactile elements (tactels). Data from such tactel arrays, such as the 16×16 grid of force-sensitive resistors [28], can be treated as a grayscale image [29]. Similar to image features in vision, tactile image features of the contact pressure profile can be computed for the estimation of a stability of a grasp. Ref. [30] detected the slippage of an object by analyzing changes of feature points of the tactile image. Data was collected from a 44×44 array of piezoelectric sensors installed on an industrial manipulator. Before the actual motion of the grasped object in a slip event, there are some feature points that remain on previous positions and points that have moved. The ratio of the immobile points to the moved points indicates the slip event.

An optical tactile sensor for slip detection based on a similar approach was delineated in [31,32]. The authors of these papers do not take into account the robot itself, i.e., robot kinematics. In contrast, [21] consider grasp stability as a probability distribution that depends on the combination of contact pressure profiles and robot configuration. They evaluated grasp stability using supervised machine learning algorithms. Processing such tactile sensing arrays at fast-enough sampling rates is a challenge. Studies in human haptic perception have shown that the change in contact state is detected by rapidly adapting mechanoreceptors that can capture only high-frequency components of the contact force [9]. The next group of methods for contact detection are inspired from this biological hint.

Dynamic vibrotactile sensors can be used to distinguish textured surfaces by dragging a rigid probe across it. The probe transmits temporal signals only. The induced vibrations can be used for surface classification. The aforementioned mechanoreceptors in human hands are the candidates for mimicking them in robot hands. Artificial tactile sensors with fast response (such as accelerometers, microphones, piezoelectric and capacitive sensors, barometers with fluid media, and recently event-based cameras [33]) provide information about vibrations at the contact point. Information about vibrations can be further used for slip detection and haptic object exploration. The dynamic response of the tactile sensing arrays was mainly limited by the sampling rate of reading devices. For instance, in [23], the sensing bandwidth was limited by the sampling rate of a commercial capacitance-to-digital converter (300 Hz for AD7147), whereas the bandwidth of a single tactile sensor can reach up 5 kHz of bandwidth [22].

Achieving stable grasp by detecting mechanical vibrations was first implemented for a hand prosthesis [34]. A more detailed review on slip detection is presented in the works of [13,35,36]. Dynamic tactile signals can be processed directly in time and frequency domains. One of the straightforward ways of detecting the slippage is to trigger it based on the presence of a high-pass-filtered tactile signal [37]. Similarly, in [38], signals of a higher bandwidth piezo-electric sensor were analyzed in time domain to detect a slip. In order to increase the performance, the robot hand was driven by pneumatic muscles rather than electric motors of PR2 robot grippers. An alternative way to detect slip is to transform tactile signals to the frequency domain and calculate the spectrum power, e.g., as in [22]. Tactile and audio (speech) signals are similar in their flow with respect to time. In this connection, the authors of [39] utilized the advances in speech recognition to discriminate object–gripper slip from object–world slip.

The aforementioned methods can be classified as passive sensors. In other words, external stimuli must be applied to detect, for example, an event of contact with an object or even human touch. In contrast, we are interested in making robots detect a subtle physical contact with the environment or even slight touch from a human operator. To achieve this goal, we leverage the low-cost Eccentric Rotating Mass (ERM) motors that are very sensitive to touch as their vibrating resonant state loses immediately. The vibration, in this case, plays a core role in triggering the tactile event, be it contact with the object during grasping or in human–robot interaction. Therefore, we present an algorithm for active sensing that increases the reliability of light contact detection. The active detection system depends on (a) a MEMS accelerometer installed in the soft fingertip of a robot gripper and (b) an ERM motor on the opposite fingertip. The contact event with an object may be considered as a condition in which the frequency of the ERM-induced vibrations deviate from the predetermined one that corresponds to the non-contact state.

The challenge that we take on in this paper is related to the field of sensor design and signal processing for contact detection. Our method is applicable in autonomous robot manipulation and human–robot physical interaction. To validate our approach, we built a test bench consisting of two fingertips and industrial robot gripper. We reinforce our experimental results by the numerical analysis of physical contacts by leveraging mass–spring–damping mechanical models. Indeed, our method is based on the investigations of the role of the mechanical impedance of the grasped object, which is applicable for any type of robot physically interacting with the world, e.g., during human–robot interaction, as briefly discussed in the experimental part of this paper.

In the following sections, we introduce our active contact sensing algorithm (Section 2.1). Then, we present an experimental setup which was designed to test our approach for various applications (Section 2.3). After that, we describe experiments and their parameters. Next, the experimental results are presented and analyzed with the appropriate statistical tests (Section 3). We conclude the paper by discussing the research issues in slip detection and our future plans for touch-driven learning approaches (Section 4).

## 2. Methods

### 2.1. Active Vibration Tactile Sensor

**Figure 1:**
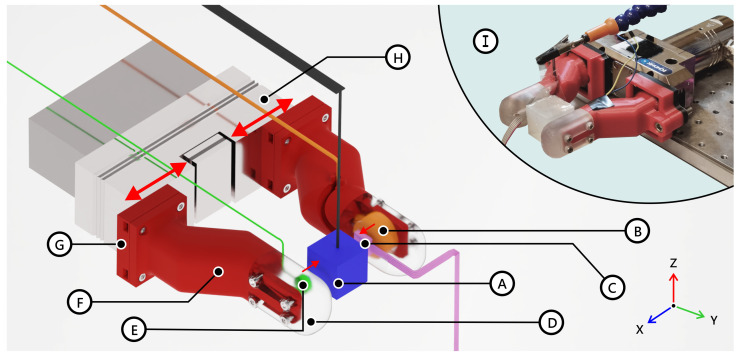
Rendered illustration for the experimental setup: the setup allows for squeezing a given object and registering the natural resonance frequencies supported in the system with the given experimental object. The object is hanging in-between the fingers of the gripper. The red arrow indicates the squeezing movement of the gripper executed along the *x*-axis; (A) object for grabbing; (B) Wittenstein F/T sensor; (C) ADXL 345 accelerometer; (D) silicone fingertips printed with FormLabs (Elastic 50A); (E) 10 mm × 3 mm coin ERM vibration motor; (F) plastic finger bone (printed with Ultimaker PLA); (G) interchangeable plastic plates for mounting of the printed fingers onto Schunk EGN robotic gripper; (H) Schunk EGN100 robotic gripper; (I) photograph of experimental setup.

Our proposed active vibration tactile sensor is illustrated in Figure 1. The sensor consists of three main parts: (i) 3D printed plastic robotic fingers (“bones”) covered with silicone rubber (“tissue” and “skin”) and mounted on Schunk EGN100 gripper, (ii) a vibration source (a coin vibration motor), and (iii) an accelerometer ADXL 345. An object can be grabbed by changing the position of the fingers attached to the gripper. We consider a scenario where the robot hand grabs a single object, which can be rigid (steel) or soft (rubber silicone). For the sake of simplicity of the experimental verification, we design the objects to have cubic shapes (blue cube in Figure 1). The vibrations are injected using the coin 10 mm × 3 mm ERM vibration motor placed inside the rubber skin of the finger (green disk). The vibrations are registered using the accelerometer placed inside another finger (pink cuboid). The squeezing force is registered using a commercial Wittenstein multi-axial force and torque sensor HEX21. The injected vibrations can propagate through the attached gripper and the squeezed object (in case of contact). Since the system consists of multiple rigid and flexible links between parts, the total actuation chain supports multiple resonance modes sensitive to any change in the chain. When the object is connected to the robot hand, the actuation chain resonance frequencies can change significantly, demonstrating the method’s high sensitivity. In the next section, we explain the changes in the resonance modes by introducing a simplified multi-body system represented using lumped components such as masses, springs, and dampers.

### 2.2. Lumped-Element Model of Robot Hand Gripper

**Figure 2:**
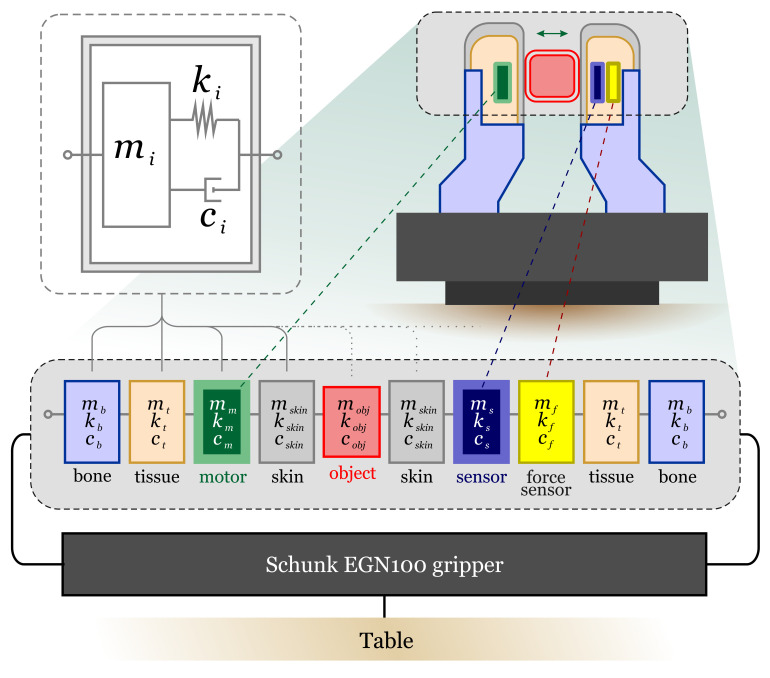
Illustration of a simplified lumped-element system used to model the vibration resonances of the robot hand gripper. In the model, the robot hand was separated into several blocks, such as parts of fingers (bone, tissue, and source), a gripper Schunk EGN100, and an object between the fingers of the robot hand. In turn, each block can be represented using lumped elements (mass, spring, and damper), as shown in the top left corner of the panel. Depending on the material and geometry, all elements of the hand are modeled using masses $m_{i}$, springs $k_{i}$, and dampers $c_{i}$. The whole system is attached to the ground (an office table).

In this section, we describe our active vibration sensor using a simplified mass–spring–damper circuit representation [40,41]. The schematic illustration of the model is depicted in Figure 2. Despite its simplicity, such a mass–spring–damper model has been successfully applied to the analysis of locomotion [42,43,44,45], demonstrating the ability of predicting important features of human body motion, including the ground reaction forces, fluctuations of the kinetic energy, etc. Therefore, we apply a lumped circuit analysis to explain the physics behind the sensitivity of our sensor and predict the optimal operating frequency given the parameters of the fabricated sensor.

The sensor is divided into several interlinked blocks that contain rigid (“bones”, gripper) and nonrigid (“tissue”, “skin”) parts, as shown in Figure 2. Each block contains mass $m_{i}$, spring $k_{i}$, and damper $c_{i}$. For simplicity, we assume a 1D motion of each block while maintaining the method’s validity. Moreover, all materials are modeled using the Kelvin–Voigt model, i.e., the spring and damper connected in parallel. Thus, the motion of the block can be described with the following equation:

(1) $$m_{i}\ddot{x}+c_{i}\dot{x}+k_{i}x=f_{i},$$

where *x* is the coordinate, and $f_{i}$ is the force acting on the block *i*. In practice, the forces between the blocks are nonlinear functions of spring deformations. However, in our model, the forces are assumed to be linear functions. The spring constants $k_{i}$ and damping coefficients $c_{i}$ are estimated using the material properties [46]. The vibrations are injected by the “motor” block, which has two nodes connected to “tissue” and “skin” blocks. One of the nodes introduces a displacement $u=u_{src}e^{i\phi }$ (where $u_{src}$ and ϕ are the amplitude and phase of the displacement) which is then the transmitted to the adjacent blocks. Moreover, the whole system is analyzed frequency domain, i.e., the frequency of displacement is changed in the range [1, 1000] Hz to observe the response of the natural resonances to the external source of vibrations. The response of our sensor to the vibration is analyzed by registering the displacement of the accelerometer’s mass $m_{a}$.

**Figure 3:**
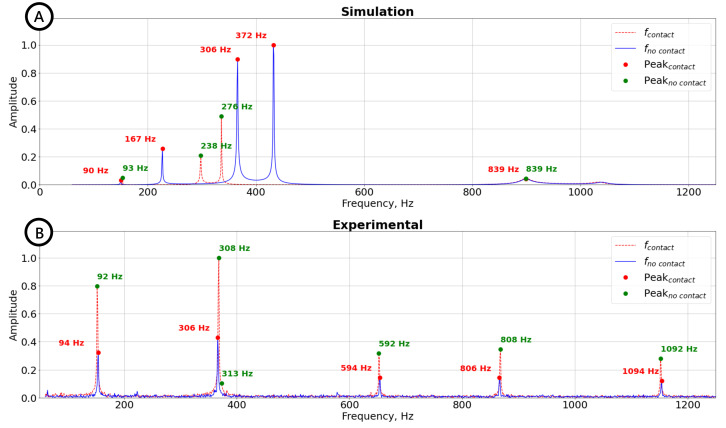
Numerical (for a simplified lumped-element model) and experimental results for the vibration resonances of the robot hand gripper. (**A**) The numerical spectra for the vibrating system without touching an object (solid blue line) and after the contact event (dashed red line). A significant displacement of resonant frequencies (marked with red and green dots for “no contact” and “contact” cases, respectively) and change in their amplitudes indicate the presence of a new element. (**B**) The experimental spectra for the vibrating robot hand system without touching an object (solid blue line) and after the contact event (dashed red line). After contact, the resonance frequencies slightly shift, while the amplitudes of the peaks change significantly (peaks for “no contact” and “contact” cases are shown with red and green dots, respectively).

First, we assume the case when the fingers do not touch the object. Therefore, for both fingers, one node of the masses of “skin” blocks is connected to a free node. By writing the motion equations for all blocks and introducing the matrices for mass M, stiffness K, and damping coefficient C, the governing equations of motion can be written in the following form:

(2) $$M\ddot{x}+C\dot{x}+Kx=f,$$

where f is an vector of forces. Such a system of differential equations can be solved analytically. However, a more practical approach is to use a numerical software. To this end, the Lumped Mechanical System Module of the commercial numerical solver COMSOL Multiphysics (COMSOL Inc., Burlington, MA, USA)^™^ was used to solve the system for our parameters. The obtained numerical spectrum for the system without touching an object (solid blue line) is plotted in Figure 3A. Several peaks corresponding to the natural resonances of the system can be observed in the plot. The vibration from the source is transmitted to the accelerometer through the gripper and other finger. Note that in the numerical experiment, we do not account the external vibration noises, which can be injected from the surrounding environment through the table.

In the next step, we consider a weak link (a low stiffness of skin $k_{skin}$) between the object and the fingers of the robot hand, emulating a light touch. Even such a slight change in the vibration propagation chain results in a significant displacement of resonant frequencies (dashed red line in Figure 3A). Therefore, the touch event can be detected by analyzing the spectral response of the vibration sensor. Moreover, the stiffness of skin and tissue depends on the applied force or the grip force. Hence, it is possible to estimate the applied force by measuring the changes in amplitudes of resonance peaks and their frequency shifts. We use these phenomena in our active vibration tactile sensor and experimentally demonstrate the touch event detection in the results section.

### 2.3. Experimental Setup

A 2-finger parallel gripper Schunk EGN100 (SCHUNK GmbH & Co. KG, Lauffen/Neckar, Germany) (Figure 1H) was used for the experimental procedure of gripping an object. Provided with precise position, velocity control, and availability, it fits perfectly into the purpose of this research. Custom artificial fingertips were modeled and produced by 3D printing to simplify the grip data collection. Each finger consists of two main parts: a plastic base (Figure 1F,G) and a silicone tip (Figure 1D). The plastic base simulates the bone in the fingertip of a real human finger and provides stability to the structure when high forces are applied. Silicone tips were produced using FormLabs Elastic 50A Resin (Formlabs, Somerville, MA, USA) to reach a remote simulation of the real elasticity of a finger combined with the ability to print complex structures needed for the project. One finger contains a 3-axis accelerometer ADXL345 (Figure 1C) for the collection of vibrations that propagate in the system (sampling frequency—2.5 kHz) and a Wittenstein HEX-21 F/T sensor (Figure 1B, sampling rate—1 kHz, recommended force range—0–50 N) used for the calibration and comparison between real and registered touch events. The other finger contains a coin ERM vibration motor (Figure 1E, generated frequency—approx. 130 Hz) for generating vibrations. Data gathering is implemented using Teensy 3.2 with package communication with ROS, where the packets of 5 samples are sent at a frequency of 500 Hz.

### 2.4. Spectral Analysis Algorithms for Contact Event Detection

**Figure 4:**
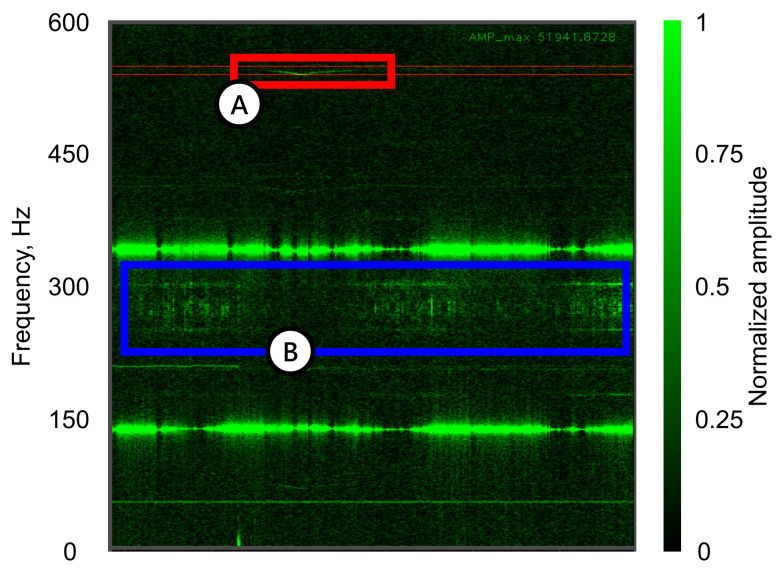
Spectrogram of a preliminary trial zoomed in on the operational frequency window. (**A**) The spectral response used for contact event detection (highlighted in red) that belongs to the frequency range of 570–580 Hz; (**B**) The noise generated by active gripper movement (highlighted in blue).

A spectrogram was used for the visual representation and preliminary identification of event trigger patterns. Data analysis and touch detection are executed via ROS framework and GUI written in Python. The spectrogram is refreshed at a rate of 100 Hz, dynamically detecting changes in the FFT of a signal acquired from the accelerometer. The trials showed that a moment when the object enters the system (gripper establishes contact with the object) could be identified by the pattern change in the specific frequency window of 570–580 Hz (Figure 4). Moreover, the proposed frequency bandwidth provides excellent isolation from the noise generated by the moving gripper. The frequency window location and width depend on the object’s properties and setup in general and can vary for different test objects. Such intricate dependence will be studied in future works. Nevertheless, for the current configuration, the proposed frequency range is optimal.

**Figure 5:**
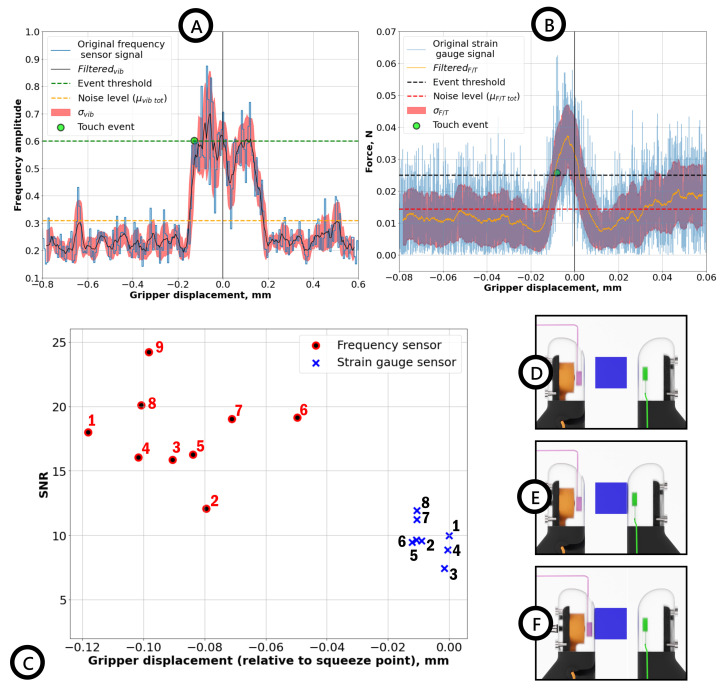
Results for object contact detection experiments. (**A**) Filtered by moving average (solid black line), raw data (solid blue line) for the amplitude change in resonance peak (≈580 Hz), and the standard deviation $\sigma_{vib}$ (semitransparent red area). The noise level and threshold are shown with horizontal orange and green lines, respectively. (**B**) Filtered (solid orange line) and raw (solid blue line) force data (HEX21) for a singular trial, with standard deviation $\sigma_{F/T}$ (semitransparent blue area) and noise level and triggering threshold (horizontal red and black lines, respectively). (**C**) Touch events for 10 trials detected by VibroTouch sensor (9 red dots) and force sensor (8 blue crosses). (**D**) No grip established, the gripper is in motion; (**E**) Case 1: actuating finger reaches contact point with the object first; (**F**) Case 2: finger containing force sensor reaches contact point with the object first.

Therefore, the touch event detection algorithm seeks the changes in peak amplitude of frequencies in the chosen range. To ensure a high accuracy rate (i.e., to prevent false detection), the trigger condition or threshold for the touch event was set to be double (obtained from preliminary tests) the noise level $2\mu_{vib}$, as shown in Figure 5A. A binary signal was used to register the touch event, where the ‘0’ value signifies the absence of touch and ‘1’ marks the occurrence of a touch event.

The trigger condition for touch events registered by the force sensor was implemented analogously. However, unlike the active vibro-sensor, post-processing of the obtained experimental data was performed by applying a moving average filter to simulate real-time smoothing (Figure 5B). The contact event trigger threshold was defined to be $1.6\mu_{F/T}$, where $\mu_{F/T}$ is the average noise level for the force sensor (≈0.015 N). Although lower than our proposed sensor, such a threshold was found to provide an optimal accuracy rate of contact detection (with false detection for lower threshold and non-detection for higher values). Below, we provide the descriptions for the experimental procedures and spectral patterns for gripper–object contact detection and grasped object–human contact detection.

#### 2.4.1. Gripper–Object Contact Detection

In the scenario of the gripper–object interaction, the experimental procedure for the detection of established contact between the gripper and an object is the following:

1. The gripper’s fingers close the gap between them with a speed of 0.01 mm/s due to the limiting maximum sampling frequency (≈30 Hz) of obtaining a proper gripper position. The dimensions of an experimental cube (side length of 17.65 mm) were used as a guide for controlling gripper displacement, with the gripper’s initial separation slightly wider than the object’s width. Therefore, the range of gripper displacement is from 0 mm to the position of the approximate dimensions of the object (where touch is supposed to happen). Hence, the maximum distance traveled by fingers is equal to 2.35 mm;
2. During this light-squeeze movement performed by the gripper, GUI collects the data from the force sensor and resonant peak amplitude changes in a specific bandwidth, actual gripper position, and touch event signal;
3. The same procedure is repeated 10 times to ensure the repeatability of the experiment, sensor’s high sensitivity, and high accuracy rate (≈90%), with corresponding post-processing of data (averages, standard deviations, etc.).

#### 2.4.2. Grasped Object–Human Contact Detection

**Figure 6:**
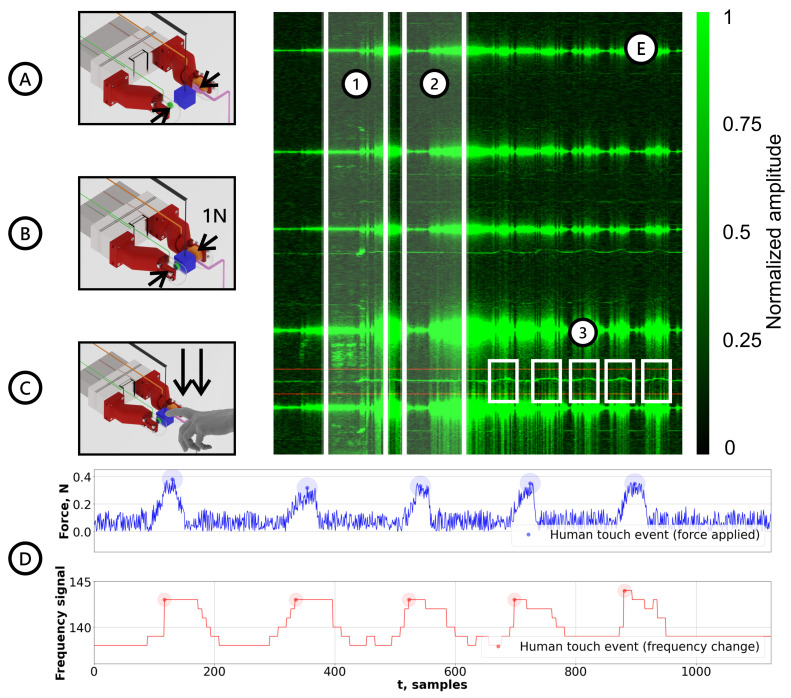
Results for the human–object contact detection experiments. (**A**) Stage 1—No grip established, the gripper is in motion; (**B**) Stage 2—Grip established with a gripping force of 1N; (**C**) Stage 3—Human applies pressure on the object grasped by the robotic gripper; (**D**-**top**) force data (HEX21) with trigger regions for human touch detection; (**D**-**bottom**) control frequency signal (HEX21) with trigger regions for human touch detection; (**E.1**) frequency spectrum region corresponding to Stage 1; (**E.2**) frequency spectrum region corresponding to Stage 2; (**E.3**) frequency spectrum region corresponding to Stage 3.

The second experimental procedure is aimed at establishing the event of human or any other external stimuli touching the object grasped by the gripper. The experimental procedure is the following:

1. Establishing the grip with a force of 1 N along the *z*-axis (Figure 6B) using force control with the feedback provided by HEX21;
2. Beginning data acquisition: collect synchronously peak frequency from the specified frequency range and force along the *y*-axis;
3. A human or an external stimulus enters the system by applying pressure on the object along the *y*-axis 5 times (Figure 6C).

In this experimental case, the preliminary trials showed that the main changes in the frequency response that are also robust to the noise coming from the gripper movements happen to be the most prominent in the bandwidth of 180–250 Hz. Other resonance frequencies, despite exhibiting similar behaviors, have not been chosen as an indicator due to weak amplitude peaks and overlap with gripper-induced noises.

As a triggering condition, analogously to the gripper–object case, a threshold for both of the signals is used in order to register the events of an external stimulus entering the system (human touch). The threshold for the force sensor was set to be equal to $\delta_{F/T}=0.3N$, as anything below this threshold can result in false detection due to sensor noise. For the vibroactive sensor, the triggering condition was set to >3% change in the resonance frequency (obtained from the initial calibration buffer).

## 3. Results

In this section, we present the experimental results for the different scenarios of the robot gripper coming into contact with objects and humans backed up by analytical model simulations.

### 3.1. Gripper–Object Contact Detection

Figure 5A shows the normalized peak amplitude (at 580 Hz) evolution obtained from the vibroactive sensor (solid blue line), filtered by the moving average in the 50 samples’ window signal (solid black line) with standard deviation $\sigma_{vib}$ (semitransparent red area). The touch event threshold and noise levels are shown with horizontal dashed orange and green lines, respectively, and the touch event with a green dot marker. Here, the filtered normalized resonance peak amplitude signal shows a significant change at the moment of contact event compared with the noise level $\mu_{vib}$ before the contact, supporting the initial claim of vibroactive sensors, exhibiting excellent signal-to-noise-ratio (SNR). On the other hand, filtered force sensor data (solid orange line) demonstrate a lower difference between the trigger signal (dashed black line) and regular noise $\sigma_{F/T}$ (dashed red line). Therefore, a delay in the contact detection with the force sensor is more prominent than the active vibrational sensor, since the force sensor requires a more significant force to be applied, which impedes detecting light touches. In fact, it registers a squeezing event rather than a touch event, and the latter could naturally happen first. For instance, Figure 5E,F show two different scenarios of contact that are more likely to occur. In one scenario (Figure 5E), the actuating finger reaches the contact point with the object before the sensing finger. In another case (Figure 5F), the sensing finger touches the object first. In both scenarios, the changes in the actuation chain are enough to trigger the VibroTouch sensor, while the force sensor requires a steady squeezing force between fingers.

Finally, the detected touch events for both sensors are shown in Figure 5C, which were obtained consecutively in 10 trials to determine light contact events. For both sensors, SNRs were calculated to describe their sensitivities quantitatively. In the case of the force sensor, the average SNR ≈ 8 is notably smaller than for the vibroactive sensor SNR ≈ 18. Moreover, this figure shows that the VibroTouch sensor detected 9 out of 10 touch events, while the force sensor detected 8 events.

### 3.2. Grasped Object–Human Contact Detection

Contact frequency changes obtained during the preliminary experiments were verified during consecutive experiments in Figure 6. Spectrogram data in Figure 6E effectively represents all of the aforementioned stages of an experimental procedure. Shaded region (1) illustrates gripper movement during force control procedure, (2) is where the force condition was achieved and brief calibration is executed for future frequency change comparison, and (3) are the five events of a human entering a vibrational system.

As it can be seen from the results on Figure 6D, the indicated threshold strongly correlates with events marked by the force sensor (chosen as "true" contact event). Thus, the sensor has proven to effectively determine the events of humans or any other external stimulus coming into contact with the system while maintaining robustness against gripper movement noise. The video of experimental human contact detection is available in Supplementary Materials.

## 4. Discussion and Conclusions

The proposed active VibroTouch sensor allows extremely sensitive contact detections, which, unlike other passive vibration sensors, does not require high energy collisions between the robot manipulator and manipulated objects. Such a sensitivity improves operational safety since low-speed robot hand—object and grasped object—human interactions are possible. The increased sensitivity of the sensor is achieved by actively injecting vibrations into the system and monitoring any change in its resonance modes, instead of relying on the vibrations produced by the collisions. Moreover, the presence of the external vibration source improves its robustness to the vibration noises that are inevitably generated by the moving parts of manipulator. In addition to the robustness to the vibration noise, the proposed sensor is potentially robust to the acceleration-induced errors in the force sensors since the natural resonances depend less on the external acceleration. However, the acceleration indifference is a subject for future investigations. Furthermore, in more precise object manipulations, when a quasi-static approximation of the gripper positions is employed, our active vibration sensor still outperforms the traditional contact detection schemes based on the estimation of the force change thanks to the resonance nature of the detection scheme. Another clear advantage of our active VibroTouch sensor over other collision detection sensors is the low cost of the components (for instance, when compared with MEMS-based force sensors) and easy implementation into existing robot manipulator setups.

On this account, it should be noted that the low-cost ERM motor in our VibroTouch sensor might not be suitable for long, continuous operation. This should not be an issue in real-world scenarios since the sensor could be driven by pulses and be off when the contact with an object is established.

The sensitivity and robustness of the proposed sensor can be further improved by modifying both vibration source and detection parts. For instance, by implementing a higher frequency source and controlling its phase component, it is possible to increase the signal isolation from noise. Moreover, by employing an analog accelerometer instead of the digital sensor, a higher sampling frequency can be achieved. Furthermore, by increasing the number of phase-controlled vibration sources, we can improve the reliability of the method against false detections. Our proposed contact detection method can be implemented in an embedded system such as a microcontroller, and, therefore, increase the dexterity of modern robotic systems.

Our proposed scheme can be extended to force and object analysis. For instance, since the stiffness of the “skin” layer of “fingers” has a nonlinear dependence on the applied force, the latter can be estimated by calibrating the sensor on known objects. Moreover, since the object properties (such as stiffness, mass, and damping coefficients, as well as the roughness of the contact surface) are parameters included in the system, they can be estimated from its spectral response as well. However, such analysis requires decoding a complex nonlinear multi-variable function, which is non-trivial task. A possible solution is to take advantage of machine-learning-based approaches that have demonstrated a tremendous potential for such data-driven tasks.

## Data Availability

The data presented in this study are available on request from the corresponding authors.

## Supplementary Materials

The following supporting information can be downloaded at: https://www.mdpi.com/article/10.3390/s22176456/s1, Video S1: Human-object Contact Detection Experiments via Active Tactile Sensing
